# Accurate Classification of Protein Subcellular Localization from High-Throughput Microscopy Images Using Deep Learning

**DOI:** 10.1534/g3.116.033654

**Published:** 2017-04-08

**Authors:** Tanel Pärnamaa, Leopold Parts

**Affiliations:** * Institute of Computer Science, University of Tartu, 50409, Estonia; † Wellcome Trust Sanger Institute, Hinxton, Cambridgeshire CB10 1SA, United Kingdom

**Keywords:** deep learning, high-content screening, machine learning, microscopy, yeast

## Abstract

High-throughput microscopy of many single cells generates high-dimensional data that are far from straightforward to analyze. One important problem is automatically detecting the cellular compartment where a fluorescently-tagged protein resides, a task relatively simple for an experienced human, but difficult to automate on a computer. Here, we train an 11-layer neural network on data from mapping thousands of yeast proteins, achieving per cell localization classification accuracy of 91%, and per protein accuracy of 99% on held-out images. We confirm that low-level network features correspond to basic image characteristics, while deeper layers separate localization classes. Using this network as a feature calculator, we train standard classifiers that assign proteins to previously unseen compartments after observing only a small number of training examples. Our results are the most accurate subcellular localization classifications to date, and demonstrate the usefulness of deep learning for high-throughput microscopy.

Microscopy images are a rich, and perhaps underutilized, source of high-throughput biological data. Endogenous proteins tagged with a fluorescent marker can report quantitative states of living cells, and help annotate gene function by recording spatial and temporal variation in localization or abundance. While biochemical assays of molecule concentrations require large lysed populations for readout, imaging can be performed on single live cells. The acquisition can be automated, producing thousands of micrographs an hour in an arrayed format. These engineering advances have paved the way for systematic screening of tagged protein collections (Huh *et al.* 2003), looking for mutant effects on protein abundance (Albert *et al.* 2014; Parts *et al.* 2014) and localization (Chong *et al.* 2015), changes in cell (Ohya *et al.* 2005) and organelle (Vizeacoumar *et al.* 2010) morphology, and assigning gene function (Farkash-Amar *et al.* 2014; Hériché 2014).

Output of a high-throughput microscopy screen has to be automatically processed (Shamir *et al.* 2010). A typical workflow consists of image normalization, cell segmentation, feature extraction, and statistical analysis; freely available tools exist that make sensible choices for each of these steps (Collins 2007; Lamprecht *et al.* 2007; Pau *et al.* 2010; Kamentsky *et al.* 2011; Wagih *et al.* 2013; Wagih and Parts 2014; Bray *et al.* 2015). Nevertheless, while the preprocessing stages of normalization and segmentation can be performed in a relatively standardized manner to obtain protein abundances, problem-specific feature extraction and statistical analysis are crucial for subcellular localization mapping. Image analysis pipelines need to carefully calculate more abstract features from raw pixel values, and select most informative ones to obtain numbers that matter in the context of the experiment at hand (Glory and Murphy 2007; Handfield *et al.* 2015). Defining the correct features can be time-consuming and error-prone, and default quantities produced by existing software are not necessarily relevant outside the domain for which they were crafted (Boland *et al.* 1998; Conrad *et al.* 2004).

Deep neural networks (LeCun *et al.* 2015; Schmidhuber 2015) have recently become popular for image analysis tasks, as they overcome the feature selection problem. Methods based on deep learning have proved to be most accurate in challenges ranging from object detection (He *et al.* 2015) to semantic segmentation (Girshick *et al.* 2014) and image captioning (Vinyals *et al.* 2015), as well as applications to biological domains (Tan *et al.* 2015; Angermueller *et al.* 2016; Rampasek and Goldenberg 2016), from regulatory genomics (Alipanahi *et al.* 2015; Kelley *et al.* 2016; Zhou and Troyanskaya 2015) to electron microscopy (Cireşan *et al.* 2012, 2013). For object identification from photos, these models already outperform humans (He *et al.* 2015). Briefly, deep networks process images through consecutive layers of compute units (neurons), which quantify increasingly complex patterns in the data, and are trained to predict observed labels. One of their main appeals is that given a large enough training set, they are able to automatically learn the features most useful for the given classification problem, without a need to design them *a priori*.

Here, we apply the deep learning paradigm to high-throughput microscopy data. We present DeepYeast, a neural network trained to classify fluorescent protein subcellular localization in yeast cells. Our network outperforms random forests trained on standard image features for determining the localization patterns, both at single cell and cell population levels, and achieves accuracies higher than previously reported. We interpret the internal outputs of the network, and find that neuron layers close to data correspond to low-level image characteristics, while deeper neurons inform of the classification state. The network can be used as a feature extractor, so that random forests trained on its output separate previously unobserved classes.

## Methods

### Data

We constructed a large-scale labeled data set based on high-throughput, proteome-scale microscopy images from Chong *et al.* (2015). Each image has two channels: a red fluorescent protein (mCherry) with cytosolic localization, thus marking the cell contour, and green fluorescent protein (GFP) tagging an endogenous gene in the 3′-end, which characterizes the abundance and localization of the protein. For ∼70% of the yeast proteome, the protein subcellular localization has been manually assigned (Huh *et al.* 2003). However, our data were acquired in a somewhat different genetic background and experimental setting, and labeling the images by eye can be error-prone. To obtain high confidence training examples, we therefore used images where (Huh *et al.* 2003; Chong *et al.* 2015) annotations agree. Our final data set comprised 7132 microscopy images from 12 classes (cell periphery, cytoplasm, endosome, endoplasmic reticulum, Golgi, mitochondrion, nuclear periphery, nucleolus, nucleus, peroxisome, spindle pole, and vacuole) that were split into training, validation, and test sets. Furthermore, segmentations from Chong *et al.* (2015) were used to crop whole images into 64 × 64 pixel patches centered on the cell midpoint, resulting in 65,000 examples for training, 12,500 for validation, and 12,500 for testing.

### Convolutional neural network

**Figure 1:**
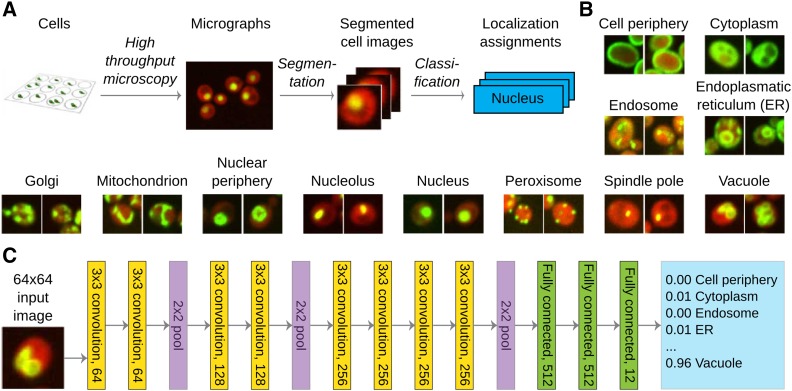
A deep neural network for protein subcellular classification. (A) Outline of the data generation and classification workflow. (B) Example pictures (two images) from each of the 12 classes (labeled above). Red fluorescence corresponds to a cytosolic marker to denote the cell, and green to the protein of interest. (C) Architecture of the “DeepYeast” convolutional neural network. Eight convolutional layers (yellow) are succeeded by three fully connected ones (green), producing the prediction (blue). All convolutional layers have 3 × 3 filters with stride 1 (filter size and number of neurons in layer label), and all pooling operations (purple) are over 2 × 2 nonoverlapping areas. ER, endoplasmic reticulum.

We trained a deep convolutional neural network that has 11 layers (eight convolutional and three fully connected) with learnable weights (Figure 1C). We used 3 × 3 patterns with step size (stride) 1 for convolutional layers, 2 × 2 aggregation regions with step size 2 for pooling layers, and rectified linear unit nonlinearities for the activation function. The number of units in the convolutional layers was 64, 64, 128, 128, 256, 256, 256, and 256, and in the fully connected layers was 512, 512, and 12. We initialized the weights using the Glorot-normal initialization technique (Glorot and Bengio 2010), and used batch normalization (Ioffe and Szegedy 2015) after each convolutional or fully connected layer, but before activation functions. For each image, per-pixel training set mean was subtracted before use. Cross-entropy loss was minimized using stochastic gradient descent with momentum of 0.9, initial learning rate of 0.1, and a mini-batch size of 100. Learning rate was divided by two after every 16,250 iterations (25 epochs). To reduce overfitting, we used weight decay of 0.0005, and dropout with rate of 0.5 for the first two fully connected layers. The models were trained for 195,000 iterations (300 epochs over full training data), and based on validation loss, the model at iteration 130,000 was chosen for all experiments. The training took 3 days on an NVIDIA Tesla K20m graphical processing unit.

### Random forest

For comparison, we trained a random forest classifier implemented in the R randomForest package (Liaw and Wiener 2002) on features from Chong *et al.* (2015) that were extracted using a CellProfiler (Bray *et al.* 2015) pipeline. In total, there are 435 different features consisting of intensity, geometric, and texture measurements on different scales, such as Haralick texture features (Haralick 1979), Gabor (Jain *et al.* 1997), and Zernike (von Zernike 1934) filters. We performed a grid search to select the number of trees to grow (50, 100, 250, 500, or 1000), the number of features to randomly sample at each split (10, 25, 50, 75, 100, 125, 150, 175, 200, 250, or 300), and the minimum size of terminal nodes (1, 2, 5, 10, or 50). Based on validation set performance, we chose 500, 100, and 1 for these hyperparameters, respectively. The final performance was evaluated on the same test data set as the neural network.

### Bootstrap confidence intervals

To obtain C.I.s on the precision and recall estimates, we resampled the test data with replacement 20,000 times, such that the number of the different class labels remained the same, and calculated the precision and recall for each class in every bootstrap sample. 2.5 and 97.5% percentiles of the resulting distribution were used as the 95% C.I.

### Protein-level classification

For both random forest and DeepYeast, we modeled the protein localization in one cell as a multinomial distribution with uninformative Dirichlet prior for the protein, and calculated the Dirichlet posterior for the protein from observations of individual cells. We used the maximum *a posteriori* estimate for protein localization. Intuitively, this approach corresponds to softly counting the number of cells assigned with each compartment and picking the compartment with the maximum count.

### Determining good quality cells

To remove a prominent source of misclassifications, we trained a random forest to discriminate between cells and noncells (*e.g.*, inappropriately segmented regions, empty areas, and imaging artifacts) based on the CellProfiler features. For each of the 12 categories, we randomly sampled without replacement 100 examples from the validation set images that were correctly classified by both DeepYeast and random forest, and labeled them as good quality cells. In addition, we inspected the validation set, and manually picked 118 noncells, resulting in a total of 1200 cell and 118 noncell images. We performed 10-fold cross-validation to choose the number of features to randomly sample at each split (2, 110, 218, 326, or 435), and whether to downsample good quality images at every bootstrap sample. Based on cross-validation performance, the final model used 100 trees, 110 features at each split, no downsampling, and achieved an accuracy of 96.7%.

### Transfer learning

To assess the generality of DeepYeast features learned in the classification task, we constructed a new data set from classes not present in the training data. The four new categories (actin, bud neck, lipid particle, and microtubule) each contained 1000 cell images for training, 500 for validation, and 1000 for testing. We fed the data into DeepYeast, and extracted the outputs of the first fully connected layer as features (every layer for the subsequent comparisons in Supplemental Material, Figure S6). We subsampled random data sets of different sizes (1, 3, 5, 10, 25, 50, 100, 250, and 500) from training data, fit a random forest classifier as implemented in the scikit-learn package (Pedregosa *et al.* 2011) to the corresponding DeepYeast and CellProfiler features, picked the best performing model on validation data, and evaluated the final performance on testing data for every data set size.

### t-SNE visualizations

We picked 1000 cells at random across all classes, processed them with the DeepYeast network, and applied t-SNE (Van der Maaten and Hinton 2008) with default parameters to the neuron outputs at the different layers.

### Data availability

The data used in this study were described in Chong *et al.* (2015), and stored in the Cyclops database presented in Koh *et al.* (2015). The single cell images that were extracted and used for training are available at http://www.cs.ut.ee/∼leopoldp/2016_DeepYeast. 

## Results

### Deep neural network to classify protein localization in yeast cell images

To perform accurate classification of protein localization in single cells and populations, we created the DeepYeast convolutional neural network learned from yeast high-throughput microscopy data generated by Chong *et al.* (2015) (Figure 1A and File S1). We used a data set comprising 90,000 cell images of 1783 proteins localized to exactly 1 of 12 cellular compartments (Figure 1B), as measured in two studies (Huh *et al.* 2003; Chong *et al.* 2015). Each image records the cytoplasmic signal in the red channel, and a tagged protein of interest in the green channel. The network consists of 11 layers (eight convolutional layers with rectified linear units, followed by three fully connected layers, Figure 1C), and a softmax output to assign one of the 12 class labels. DeepYeast’s parameters (over 10,000,000 in total) were learned in the Caffe framework (Jia *et al.* 2014), using stochastic gradient descent with momentum (*Materials and Methods*).

### Accurate classification of protein localization in single cells and populations

We first compared the performance of DeepYeast trained on raw pixel values to random forests (Breiman 2001) trained on 435 features extracted using CellProfiler (Bray *et al.* 2015) by Chong *et al.* (2015). We fitted the models on 72% of the single cell images using a range of parameter settings, picked the one with highest accuracy on another 14% of the images, and quantified its performance on the remaining 14% (*Materials and Methods*). No protein had a cell image in more than one of the training, testing, and validation folds.

**Figure 2:**
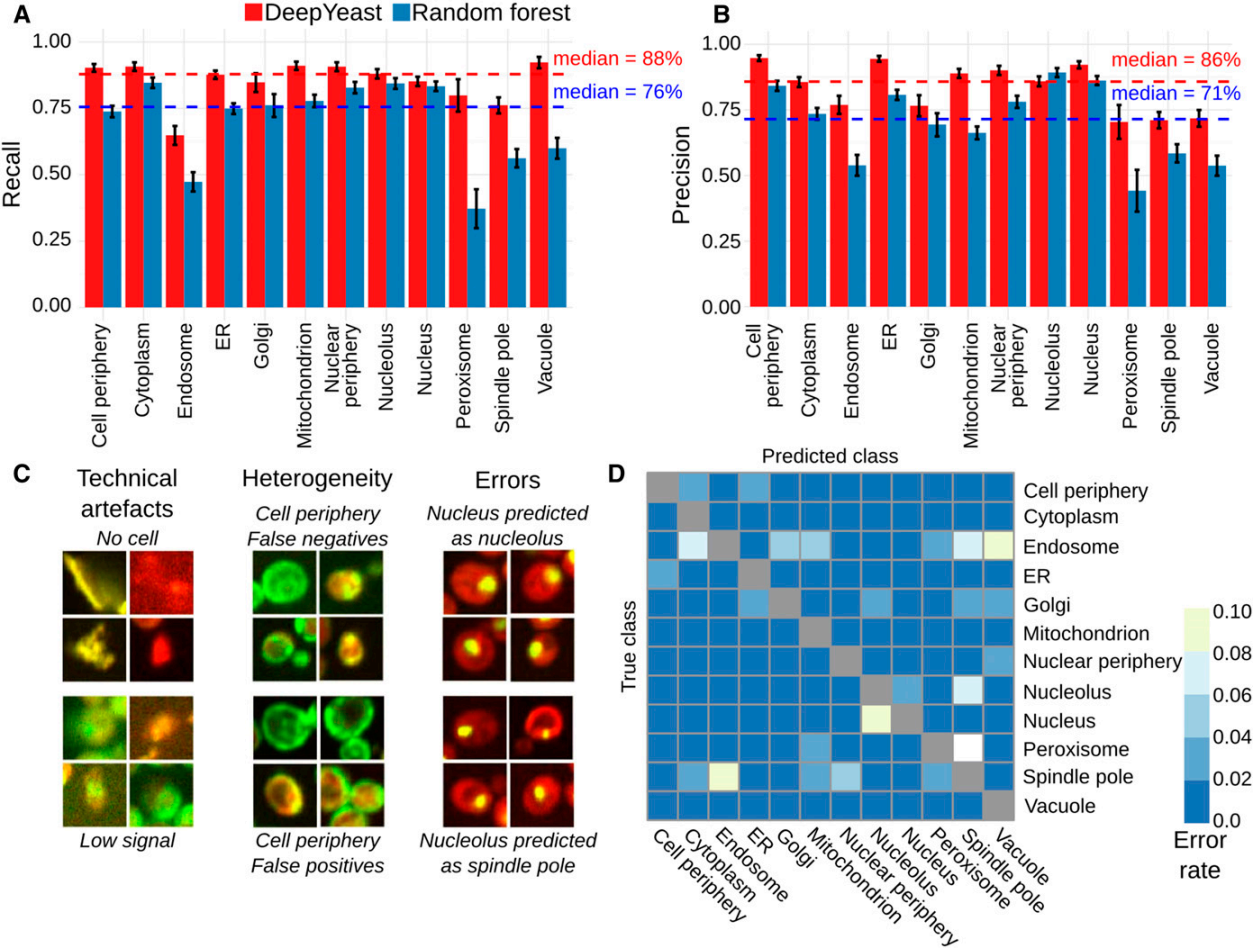
Cellular compartment classification accuracy. (A) DeepYeast outperforms random forests in classification precision. Recall (*y*-axis) for the 12 subcellular compartments (*x*-axis) for DeepYeast (red) and random forest (blue) classifiers. The dashed lines denote medians across compartments. The error bars denote the 95% C.I. from 20,000 bootstrap samples (Table S2). (B) Same as (A), but for precision on the *y*-axis. (C) Example classification mistakes stemming from technical issues (left) due to low signal (bottom left) or no cell (top left), population heterogeneity (middle) resulting in false positives (top middle) and false negatives (bottom middle), as well as frequent model errors (right) of classifying nucleus as nucleolus (top right), or nucleolus as spindle pole (bottom right). (D) Confusion matrix of DeepYeast classification. Error rates from the true (*y*-axis) to falsely predicted (*x*-axis) compartments. ER, endoplasmic reticulum.

The deep neural network achieved classification accuracy of 87% [10,839/12,500 cells, Cohen’s κ (Cohen 1960) = 0.85], compared to 75% (9375/12,500, Cohen’s κ = 0.72) for random forests (Table S1, File S2, and File S3). DeepYeast outperformed random forests for each class in recall (Figure 2A) and precision for all compartments except the nucleolus (Figure 2B). The random forest performance is concordant with previous results for single cell classification on the same data set [70% accuracy (Kraus *et al.* 2015)], which were obtained using an extended set of classes.

Mistakes occurred for each cellular compartment. Some of the errors were due to technical problems with the image, caused by low signal intensity, artifacts, or lack of proper cell (Figure 2C, left). While our results were generally robust to such noise in the input data, we further trained a classifier to distinguish good quality cell images from the CellProfiler features (*Materials and Methods*) and filtered out data deemed to have technical issues, as has been done in previous applications (Chong *et al.* 2015). After removing 1440 data points classified as noncell (12%), DeepYeast accuracy increased to 91% (10,080/11,060), and random forests to 79% (8756/11,060). Some remaining errors could be ascribed to labeling mistakes from contamination or population heterogeneity (Figure 2C, middle), causing the training data label to be discordant with the observed protein distribution in the cell. In the rest of the cases, DeepYeast classified the protein to the wrong compartment (*e.g.*, Figure 2C, right).

The most difficult localizations to classify were endosome (recall 65%, 447 correct out of 689), spindle pole (76%, 595/781), peroxisome (80%, 131/164), Golgi (85%, 324/382), and nucleus (85%, 1386/1627). Endosomes, spindle poles, peroxisomes, and Golgi are mainly represented by varying numbers of puncta, which are not visible in all cells and may obscure each other, making them difficult to distinguish. Indeed, the most frequent misclassifications (Figure 2D and Figure S1) were peroxisome to spindle pole (11%; 18 of 164 peroxisome cell images), and endosome to vacuole (8%, 56/689). Another recurring error was designating nucleolar proteins as nuclear (4%, 45/1263), both of which are large round patches. Random forests had additional common mistakes, but the most frequent misclassifications were shared with DeepYeast, reflecting the general difficulty in distinguishing punctate and patch-like patterns in a single cell (File S4 and File S5).

So far, we looked at individual cells, and classified the localization pattern of the fluorescent signal. Next, we asked how well we can infer the cellular compartment of a protein from all the cell images acquired for it. We assigned the localization class of each protein as the most probable class according to the posterior probability calculated from aggregating single cell data (*Materials and Methods*). Using this combined estimate, we achieved 99% classification accuracy (279/282) on the held-out test proteins, for which no single cell images were used for training. Two of the errors were nuclear proteins misclassified as nucleolar, with seven and nine cell images observed, respectively. The remaining error occurred for a protein with a single imaged cell. Thus, further requiring at least 10 cells to be recorded for each protein, the accuracy increased to 100% (222/222). Random forests were 95% accurate (269/282) on complete test data, and 96% accurate (214/222) when at least 10 cells were measured (Figure S2). As a baseline, Chong *et al.* (2015) reported per-protein accuracies of 50–90% (Chong *et al.* 2015) depending on the class on an overlapping data set, using the (Huh *et al.* 2003) annotation as a gold standard, and support vector machine ensemble classification. While, to our knowledge, human accuracy on these or similar images has not been assessed directly, experts assign compartments to human proteins with over 80% accuracy (Murphy *et al.* 2003; Kraus *et al.* 2015). All these performances are below what we report here, but as the data sets are not identical, direct comparisons should be interpreted with caution.

### Neural network outputs are interpretable

**Figure 3:**
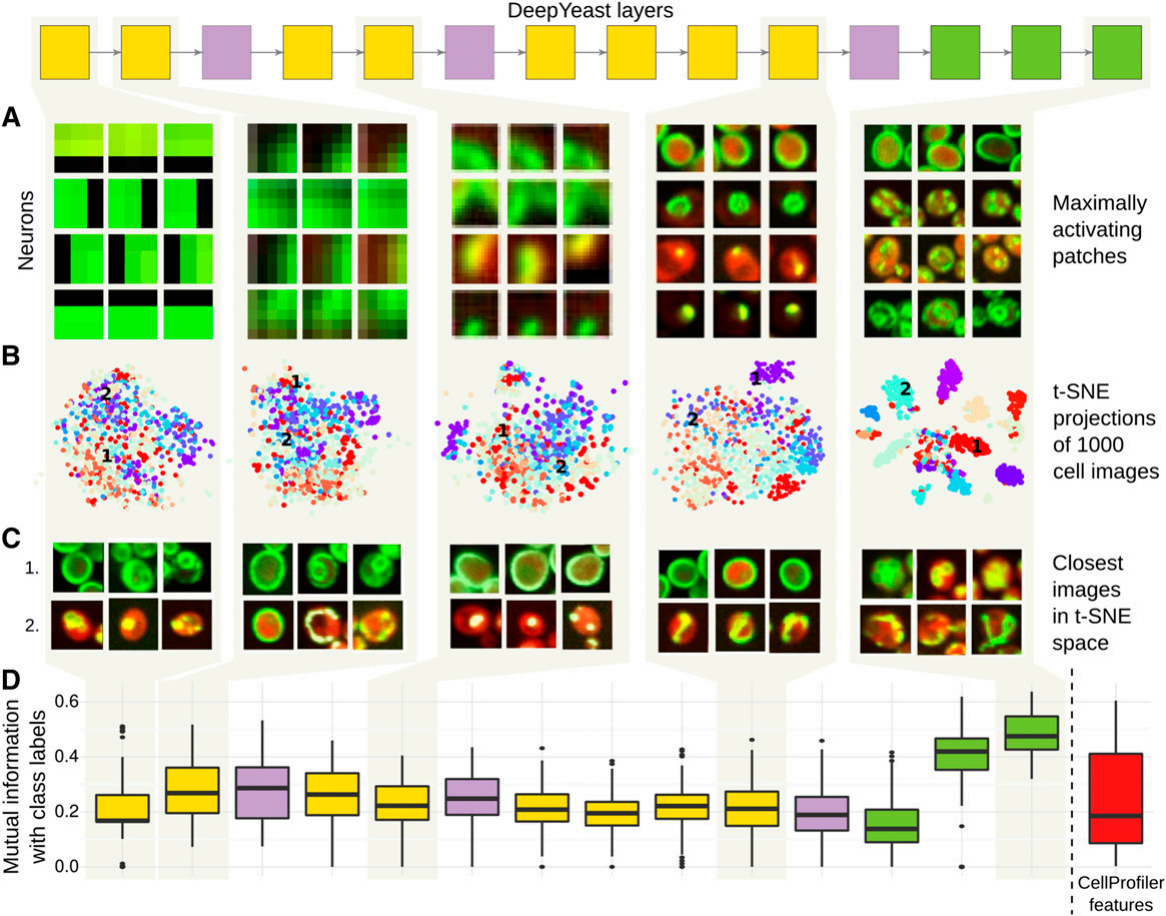
Visualization of the network features at different layers. Interpreting the first, second, fourth, eighth, and eleventh layers of DeepYeast (box diagram, top, see also Figure 1C). (A) Image patches that maximize some neuron output. For each of the layers, four neurons (*y*-axis) and image parts (*x*-axis) corresponding to a block of pixels that feed into them for maximum activation are shown. (B) 2D visualizations using the t-SNE algorithm (Van der Maaten and Hinton 2008). 1000 random images were fed through the network, hidden layer outputs were extracted, and the t-SNE algorithm was used to project the high-dimensional representations into two dimensions. The points are colored based on the true class categories. (C) Three closest images (*x*-axis) to two chosen points [1 and 2 in (B), *y*-axis] in the two-dimensional t-SNE projection space. (D) Distribution of mutual information (*y*-axis) between the multinomial class probability and discretized neuron outputs for each layer (left to right), as well as CellProfiler features (rightmost box, red).

Neural network models are often viewed as black boxes that are difficult to interpret. To gain intuition about DeepYeast features that aid prediction, we explored the characteristics of learned weights and neuron outputs. We first selected images and image patches that maximize or minimize activations of individual neurons, thus matching their weight pattern well (Figure 3A). The first layers of neurons are closest to data, with small receptive fields made up by a limited number of pixels, and thus capture local, small-scale image characteristics. As an illustration, four neurons selected in the first layer were maximally activated by image patches containing edges (Figure 3A, left column), second layer neurons by patches with corners and lines, and third and fourth layers with more complex shapes (Figure 3A, middle columns). Neurons in deeper layers represent combinations of low-level features. The maximally activating patches for selected neurons started resembling class characteristics, such as punctate patterns, membrane structures, and large patches (Figure 3A, rightmost column). While these commonalities are suggestive, and may indicate the patterns that are learned by the network, they can also be due to uninteresting technical reasons, and should thus be interpreted with caution.

Next, we applied t-SNE (Van der Maaten and Hinton 2008), a tool to visualize high-dimensional data in two dimensions, on different layers of DeepYeast outputs from 1000 randomly sampled images, and added compartment information in colors (Figure 3B and Figure S3). The classes overlap substantially for lower layer outputs, while deeper layers that make use of fully connected network structure increasingly separate the localizations, such that nearby points correspond to the same class (Figure 3C). We also asked which neuron outputs are correlated to the CellProfiler features and class membership. To do so, we calculated the strongest Pearson correlation coefficient to a CellProfiler feature (as extracted by Chong *et al.* (2015)), as well as the largest mutual information with a class label for each unit output. The deep activations informed class labels (Figure 3D), while shallow ones were more highly correlated to CellProfiler features and Gabor filters (Figure S4).

### DeepYeast can be used as a feature extractor

Classification of new localization classes requires creating new training sets, and if the pattern is rare, obtaining the necessary images is difficult and time-consuming. Further, while applying an existing network to new data can be simple, retraining it requires substantial effort. This motivates repurposing of trained networks as extractors of informative features, which can then be used as inputs to traditional models (Donahue *et al.* 2013; Razavian *et al.* 2014).

**Figure 4:**
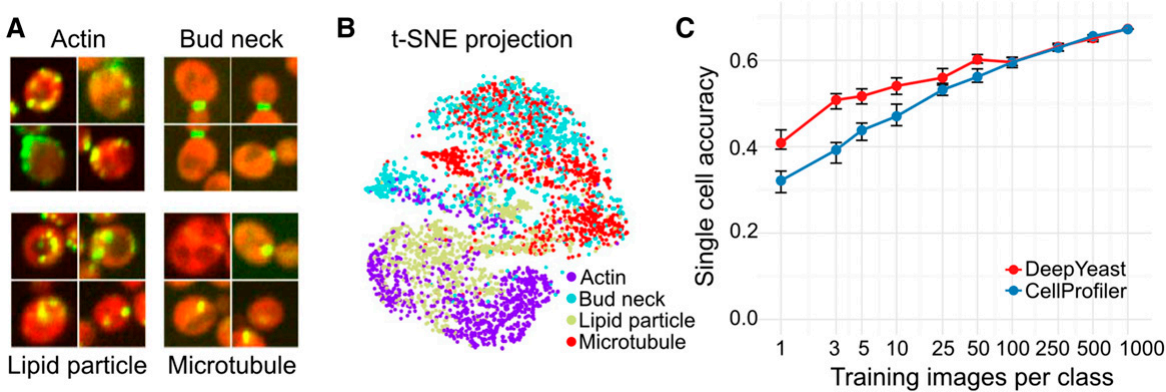
Transfer learning works. (A) Four example images of each of the additional analyzed classes. (B) Applying t-SNE to the network outputs of the additional data (see also Figure 3B) and coloring the points according to the classes demonstrates separation of new compartments based on features trained for classifying other localizations. (C) Classification accuracy on held-out data (*y*-axis) for different number of training images (*x*-axis) for DeepYeast outputs (red) or CellProfiler features (blue) used as inputs to a random forest. The error bars denote a 95% C.I. from 20,000 bootstrap samples.

We tested whether a network trained on a large amount of data can be used to distill image information that is useful for distinguishing previously unobserved compartments as well. We processed images corresponding to four new challenging classes (actin, bud neck, lipid particle, and microtubule; Figure 4A and File S6.) with DeepYeast, and calculated outputs from the first fully connected layer as features. The class labels were not independent of the features even without additional training (Figure 4B), indicating that the network extracted informative signals from the data. Next, we trained a random forest classifier on the calculated features, using an increasing number of training images. The classifiers using neural network features outperformed ones using CellProfiler features for small training set sizes (Figure 4C and Figure S5), and accuracy increased further with additional data. However, the overall accuracy on these classes remained lower than others due to their punctate pattern. We repeated the experiment on the outputs of all DeepYeast layers, and found that they do not distinguish new classes equally well. In particular, the classifiers trained on the deepest convolutional layers outperformed models trained on CellProfiler features and other layers for larger training set sizes (Figure S6). This supports previous reports that intermediate layer outputs can be more useful in a new domain, as they capture general enough features on one hand, but are not overspecialized to the trained task on the other (Yosinski *et al.* 2014).

DeepYeast was trained on proteins that predominantly localize to a single compartment. Finally, we confirmed that proteins spread between multiple classes can be accurately inferred as such. From the set of proteins assayed by Chong *et al.* (2015) not used in our analyses so far, we selected the ones manually annotated to belong to both nucleus and cytoplasm, and calculated their posterior class probabilities. As expected, cytoplasmic and nuclear classes had high posterior probability, and were the two most probable classes in 21/24 cases, and in the top three for the remaining cases. The per-gene posterior probability of compartment assignment can further be interpreted as the frequency of cells for which the protein resides in the compartment; the model is not forced to make a sharp decision and assign each gene to a single location.

## Discussion

We have demonstrated that DeepYeast, an 11-layer convolutional neural network, can achieve classification accuracy of 91% for individual cells over 12 subcellular localizations, and 100% for proteins when entire cell populations of at least moderate size are considered. Far from being a black box, the internal outputs that DeepYeast produces can be visualized and interpreted in terms of image characteristics. The pretrained network functions as a feature extractor to successfully distinguish previously unseen classes, and infer mixtures of compartments in a population.

The classification errors mostly occurred between compartments that are also difficult to distinguish by eye. The various numbers of puncta in peroxisomes, spindle poles, and endosomes can look like each other, or not be present at all. Nucleus and nucleolus are patches of similar size; when the characteristic crescent shape of the nucleolus is not showing, it is also difficult to distinguish from the nuclear marker. Overall, the single cell accuracy of 91% is approaching the protein compartment assignment performance of previous reports (Boland and Murphy 2001; Murphy *et al.* 2003; Conrad *et al.* 2004; Kraus *et al.* 2015), and the remaining errors are often borderline cases, for which classification is difficult even for trained humans (File S4 and File S5). Nevertheless, when at least 10 individual cells were measured, the correct cell classifications dominated the errors, and all test proteins were assigned to the right compartment in held-out data.

The success of deep neural networks in image analysis relies on architectures that encapsulate a hierarchy of increasingly abstract features relevant for classification, and plentiful training data to learn the model parameters. While first applications used a smaller number of layers (Boland *et al.* 1998) and mostly operated on precalculated features (Boland *et al.* 1998; Boland and Murphy 2001; Conrad *et al.* 2004; Chen *et al.* 2007), pixel level analyses gave good results (Danckaert *et al.* 2002), especially using the latest training methods (Cireşan *et al.* 2013; Kraus *et al.* 2015). Subcellular localization is defined by spatial variation on different length scales, from single small dots to extended thin membranes. Quantification of this covariance structure is thus important for accurate modeling, but deriving the right features for it requires mathematical sophistication and computational crafting (Handfield *et al.* 2015). The convolutional layers in the neural network are agnostic to the location of the signal in the image, and take inputs from progressively larger patterns, thus capturing spatial correlations of increasingly wide range in a data-driven manner.

The choice of model architecture was guided by previous results and practical considerations. Even with modern GPUs, the end-to-end training of a deep neural network is computationally intensive; therefore, we did not attempt to evaluate the influence of model architecture on the results. Three fully connected layers and the use of 3 × 3 filters has previously been shown to offer a rich enough parametrization to capture interesting feature combinations, outperforming alternatives in standardized tasks (Simonyan and Zisserman 2014). The eight convolutional layers with three 2 × 2 pooling operations gives features that span the entire cell image, at which scale we expected the organelle characteristics to be reflected. Finally, the architecture we chose was the largest that could fit in the memory of the graphical processing unit we used.

DeepYeast can be reused for other image analysis experiments with the same marker proteins and magnification, or trained further for specific applications. We demonstrated that a pretrained model can be applied for both classifying previously unseen compartments and inferring mixtures of localization patterns. The usual classification implementations do not always provide models that are easy to reuse. We envision a repository of networks trained on various bioimage compendia that can be downloaded and employed as out-of-the-box feature calculators, or fine-tuned with additional data to obtain niche-specific results, provided access to the necessary infrastructure is available. Similar resources already exist in other domains, and are being pioneered in bioimage analysis as well (Kraus *et al*. 2017).

While our DeepYeast network outperformed the random forest alternative, and achieved accuracies better than reported before, the direct comparisons must be interpreted with care. We used a clean training set of proteins localized to a single compartment as was done in previous work (Chong *et al.* 2015; Kraus *et al.* 2015), but as the training data do not match completely, the performance differences may partly be due to the data set composition. Both Chong *et al.* (2015) and Kraus *et al.* (2015) relied on information from segmented cells for best classification performance; we considered only patches known to contain a cell without pixel-level segmentation information. This circumvents the need for very accurate segmentation pipelines, and indeed, centers of cells can also be derived from additional markers, *e.g.*, histone tags in the nucleus (Parts *et al.* 2014) that are cleanly separated, and therefore much easier to segment than entire cells.

Deep neural networks have proved their value in extracting information from large-scale image data (Krizhevsky *et al.* 2012; He *et al.* 2015; LeCun *et al.* 2015; Ronneberger *et al.* 2015). It would be unreasonable to believe that the same will not be true for high-throughput microscopy. Adaptation of the technology will depend on the ease with which it is deployed and shared between researchers; to this end, we have made our trained network freely available. The utility of these approaches will increase with accumulation of publicly shared data, and we expect deep neural networks to prove themselves a powerful class of models for biological image and data analysis.

## Supplementary Material

Supplemental material is available online at www.g3journal.org/lookup/suppl/doi:10.1534/g3.116.033654/-/DC1 and the corresponding file descriptions in File S7.
