# Supplementary figures and images for: Accurate Classification of Protein Subcellular Localization from High-Throughput Microscopy Images Using Deep Learning
Source: G3 (Bethesda). 2017 Apr 8;7(5):1385–92. doi: 10.1534/g3.116.033654 (PMC5427497; doi:10.1534/g3.116.033654)

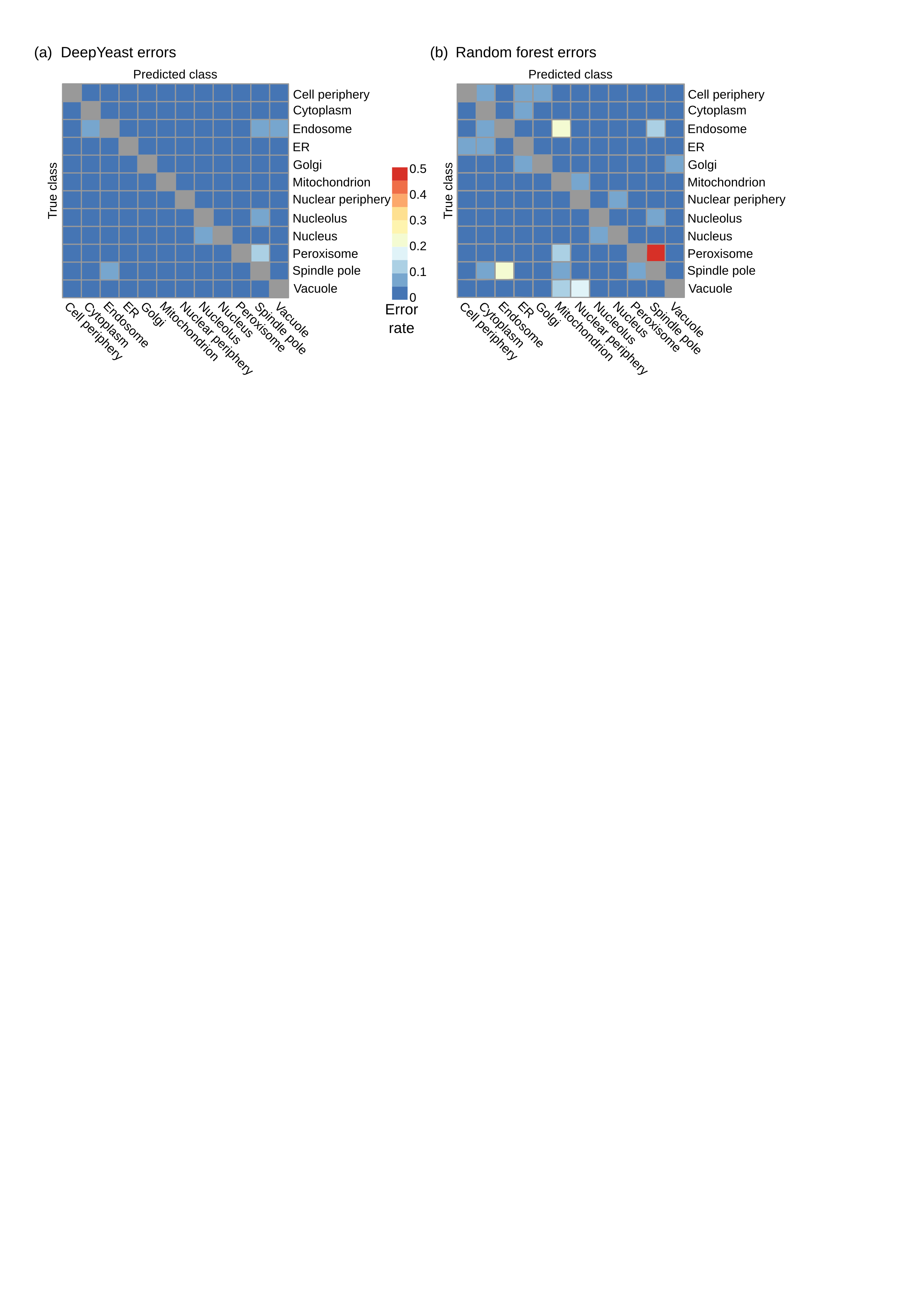

Supplement: Supplementary file 1 [file 1385FigureS1.tif]

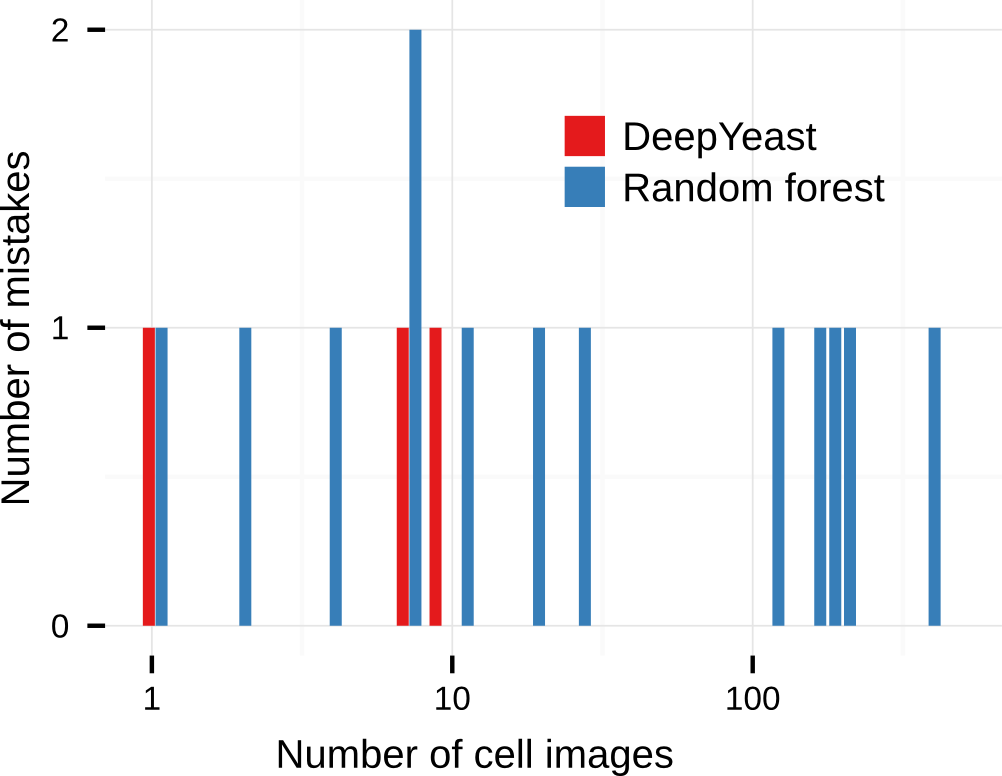

Supplement: Supplementary file 2 [file 1385FigureS2.tif]

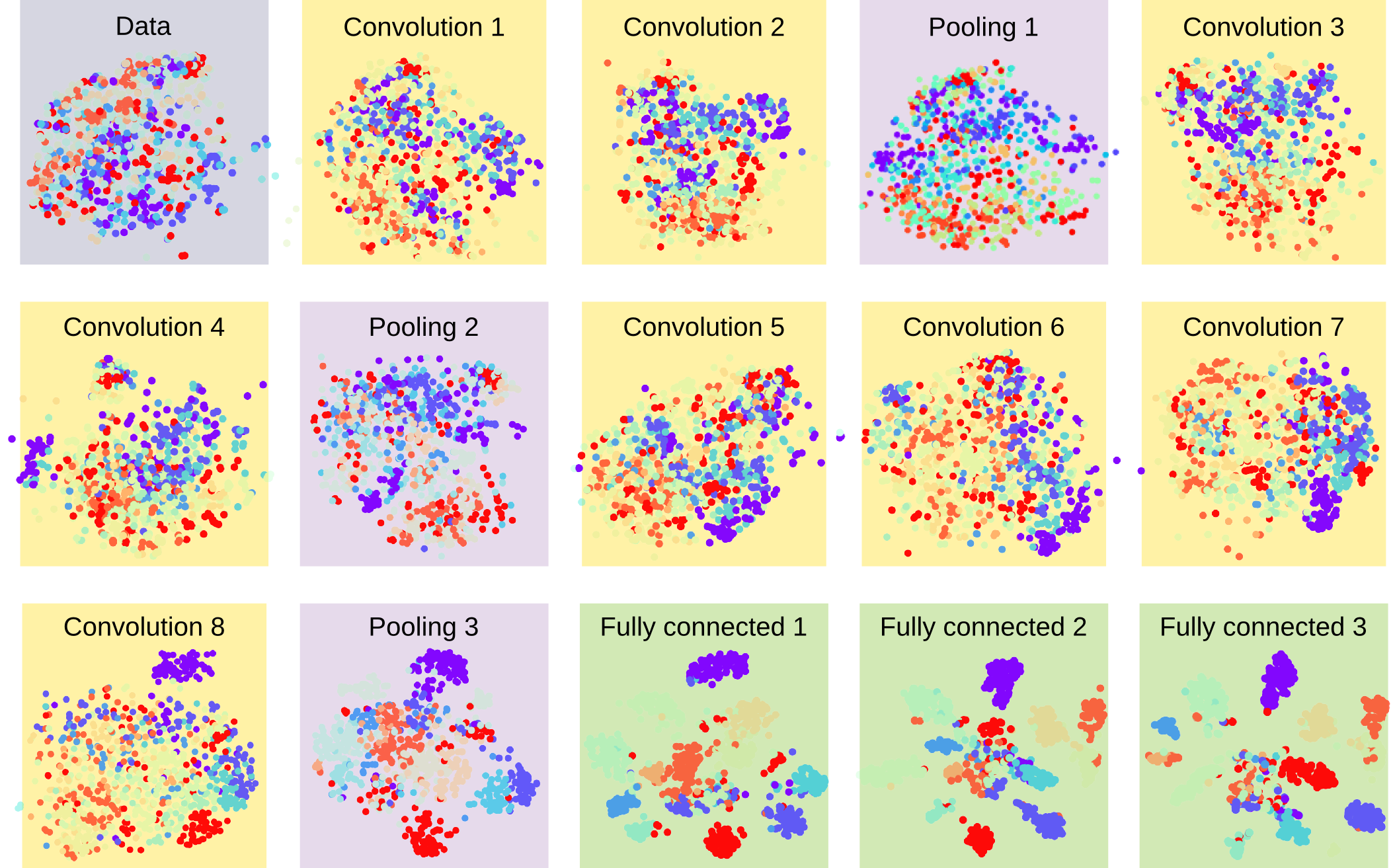

Supplement: Supplementary file 3 [file 1385FigureS3.tif]

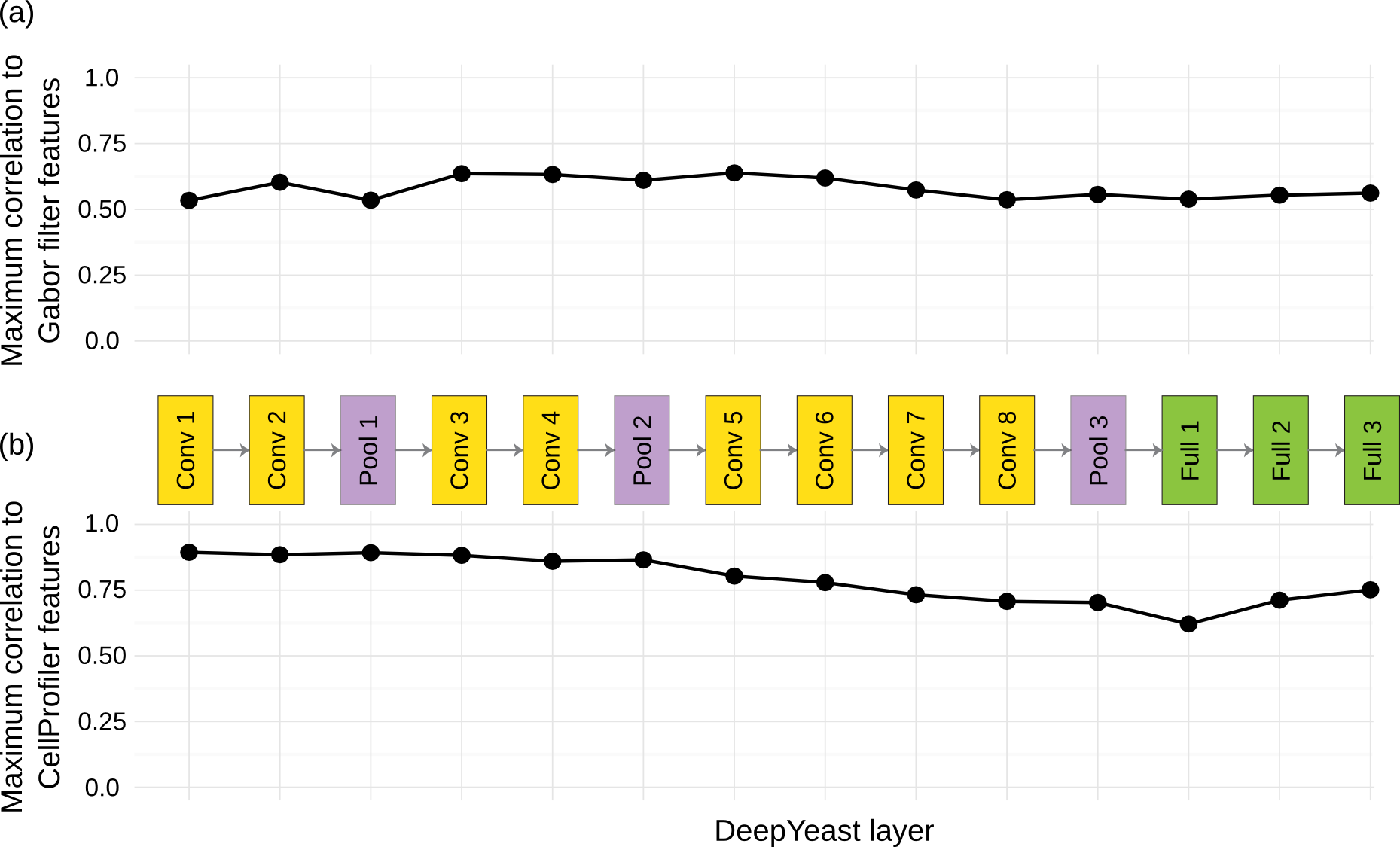

Supplement: Supplementary file 4 [file 1385FigureS4.tif]

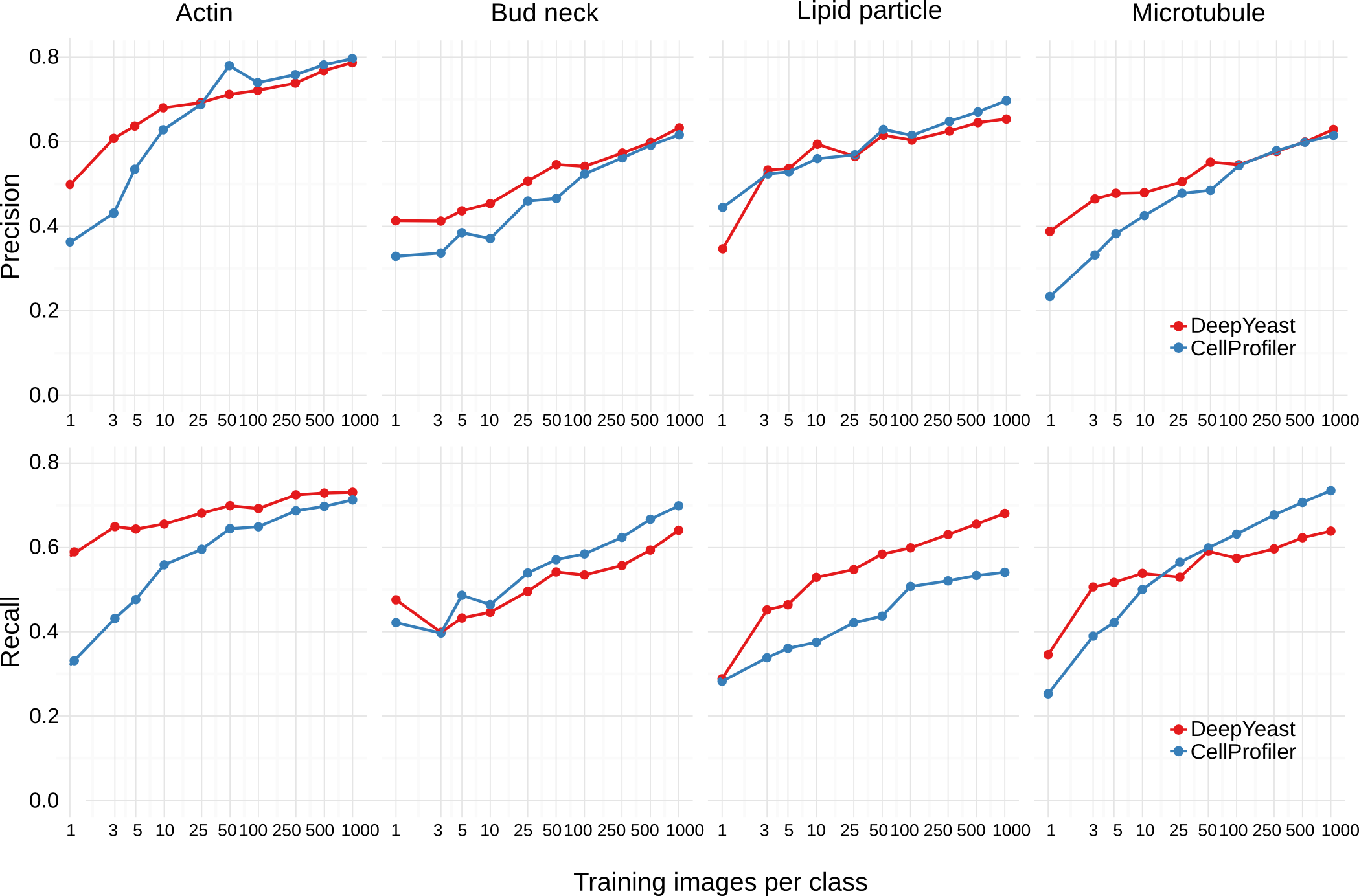

Supplement: Supplementary file 5 [file 1385FigureS5.tif]

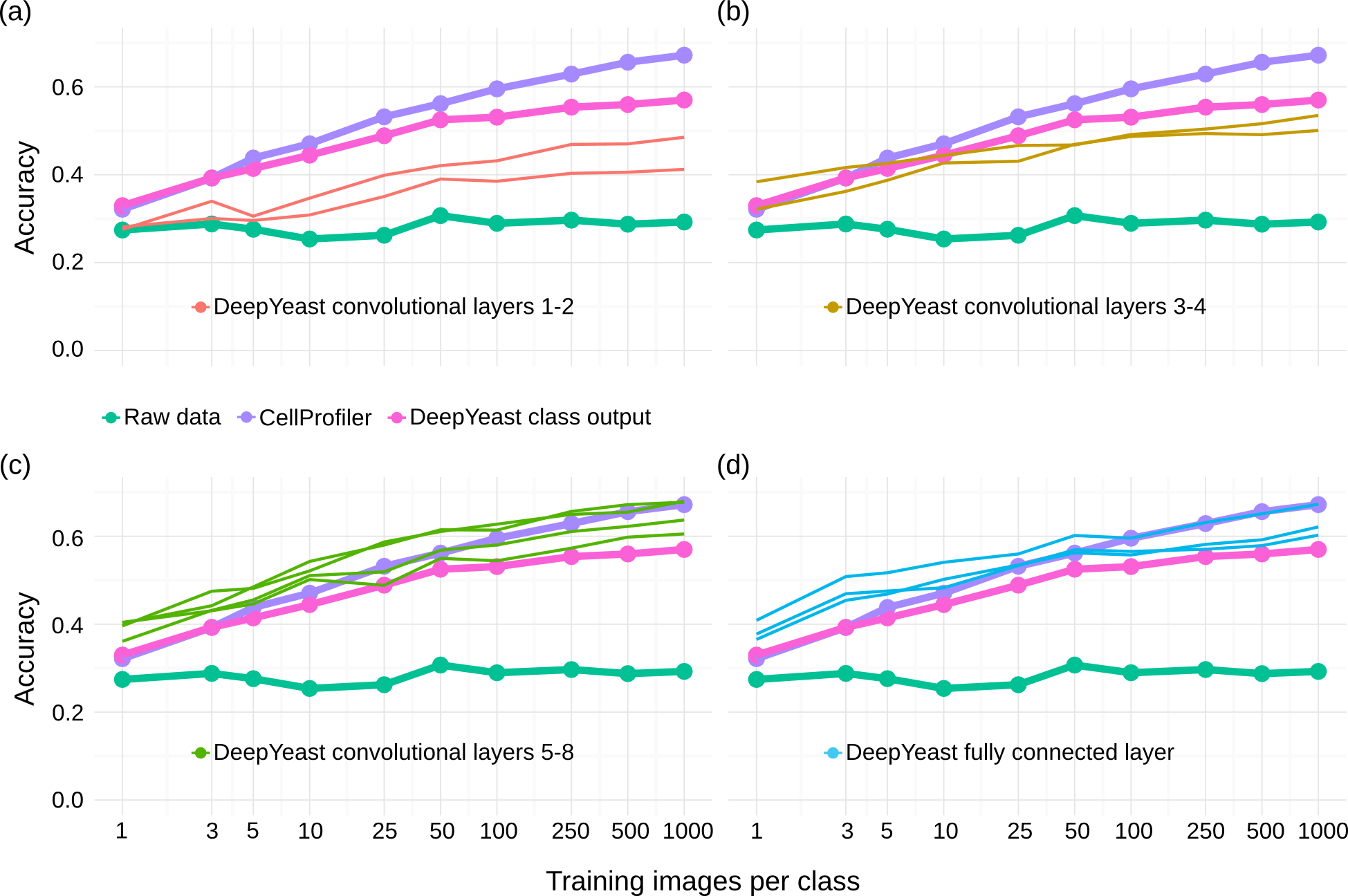

Supplement: Supplementary file 6 [file 1385FigureS6.tif]
