# Supplementary material for: Accurate Classification of Protein Subcellular Localization from High-Throughput Microscopy Images Using Deep Learning
Source: G3 (Bethesda). 2017 Apr 8;7(5):1385–92. doi: 10.1534/g3.116.033654 (PMC5427497; doi:10.1534/g3.116.033654)
Supplement: Supplementary file 9 [file 1385TableS2.docx]

Table S2. Precision and recall bootstrap confidence intervals for DeepYeast and random forest on test data. (.xlsx, 54 KB)

[http://www.g3journal.org/lookup/suppl/doi:10.1534/g3.116.033654/-/DC1/TableS2.xlsx](http://www.g3journal.org/lookup/suppl/doi:10.1534/g3.116.033654/-/DC1/TableS1.xlsx)
