# Supplementary material for: Accurate Classification of Protein Subcellular Localization from High-Throughput Microscopy Images Using Deep Learning
Source: G3 (Bethesda). 2017 Apr 8;7(5):1385–92. doi: 10.1534/g3.116.033654 (PMC5427497; doi:10.1534/g3.116.033654)
Supplement: Supplementary file 11 [file 1385FileS1.zip › FileS1.html]

Class frequencies


### Class frequencies

### Protein counts per class

Each protein was imaged four times.

| Class | Training images | Validation images | Test images |
| --- | --- | --- | --- |
| cell periphery | 27 | 4 | 7 |
| cytoplasm | 499 | 100 | 108 |
| endosome | 7 | 2 | 2 |
| er | 92 | 25 | 29 |
| golgi | 8 | 1 | 2 |
| mitochondrion | 198 | 58 | 45 |
| nuclear periphery | 22 | 5 | 5 |
| nucleolus | 39 | 6 | 7 |
| nucleus | 283 | 62 | 69 |
| peroxisome | 7 | 1 | 1 |
| spindle pole | 21 | 5 | 2 |
| vacuole | 21 | 8 | 5 |

### Sanity check for class overlap

```
## [1] "# test imgs in training set: 0"
```

```
## [1] "# val imgs in training set: 0"
```

```
## [1] "# test imgs in validation set: 0"
```

### Class examples (from training set)

```
## [1] "cell periphery"
```

```
## [1] "cytoplasm"
```

```
## [1] "endosome"
```

```
## [1] "er"
```

```
## [1] "golgi"
```

```
## [1] "mitochondrion"
```

```
## [1] "nuclear periphery"
```

```
## [1] "nucleolus"
```

```
## [1] "nucleus"
```

```
## [1] "peroxisome"
```

```
## [1] "spindle pole"
```

```
## [1] "vacuole"
```

### Scaled class examples (from training set)

```
## [1] "cell periphery"
```

```
## [1] "cytoplasm"
```

```
## [1] "endosome"
```

```
## [1] "er"
```

```
## [1] "golgi"
```

```
## [1] "mitochondrion"
```

```
## [1] "nuclear periphery"
```

```
## [1] "nucleolus"
```

```
## [1] "nucleus"
```

```
## [1] "peroxisome"
```

```
## [1] "spindle pole"
```

```
## [1] "vacuole"
```
