# Supplementary material for: Accurate Classification of Protein Subcellular Localization from High-Throughput Microscopy Images Using Deep Learning
Source: G3 (Bethesda). 2017 Apr 8;7(5):1385–92. doi: 10.1534/g3.116.033654 (PMC5427497; doi:10.1534/g3.116.033654)
Supplement: Supplementary file 13 [file 1385FileS2.zip › FileS2.html]

Model: DeepYeast


### Model: DeepYeast

### Accuracy

DeepYeast accuracy on validation data was 0.869, and on test data 0.867.

### Accuracy per category

| Class | Precision | Number of cell images | Correct calls | Recall | Accuracy |
| --- | --- | --- | --- | --- | --- |
| cell periphery | 0.948 | 1569 | 1417 | 0.903 | 0.982 |
| cytoplasm | 0.857 | 1276 | 1158 | 0.908 | 0.975 |
| endosome | 0.769 | 689 | 447 | 0.649 | 0.970 |
| er | 0.945 | 1755 | 1539 | 0.877 | 0.976 |
| golgi | 0.766 | 382 | 324 | 0.848 | 0.987 |
| mitochondrion | 0.889 | 1243 | 1132 | 0.911 | 0.980 |
| nuclear periphery | 0.901 | 1164 | 1056 | 0.907 | 0.982 |
| nucleolus | 0.859 | 1263 | 1112 | 0.880 | 0.973 |
| nucleus | 0.922 | 1627 | 1386 | 0.852 | 0.971 |
| peroxisome | 0.704 | 164 | 131 | 0.799 | 0.993 |
| spindle pole | 0.711 | 781 | 595 | 0.762 | 0.966 |
| vacuole | 0.718 | 587 | 542 | 0.923 | 0.979 |

Across classes, the mean and median precision were 0.83 and 0.86 respectively, recall 0.85 and 0.88, and accuracy 0.98 and 0.98.

### Confusion matrix

Each element \(M\_{ij}\) in the confusion matrix \(M\) shows the number of observations from class \(i\) which were predicted as class \(j\). The confusion matrix in the figure below has been normalized by class support size (each row has been divided by the number of elements in that class).

|  | cell periphery | cytoplasm | endosome | er | golgi | mitochondrion | nuclear periphery | nucleolus | nucleus | peroxisome | spindle pole | vacuole |
| --- | --- | --- | --- | --- | --- | --- | --- | --- | --- | --- | --- | --- |
| cell periphery | 1417 | 52 | 8 | 43 | 4 | 7 | 8 | 4 | 6 | 0 | 3 | 17 |
| cytoplasm | 18 | 1158 | 15 | 17 | 2 | 8 | 6 | 7 | 7 | 0 | 25 | 13 |
| endosome | 2 | 44 | 447 | 5 | 30 | 28 | 9 | 0 | 5 | 14 | 49 | 56 |
| er | 52 | 31 | 12 | 1539 | 32 | 21 | 21 | 3 | 1 | 6 | 6 | 31 |
| golgi | 1 | 2 | 0 | 9 | 324 | 5 | 5 | 11 | 0 | 1 | 12 | 12 |
| mitochondrion | 1 | 10 | 4 | 5 | 9 | 1132 | 9 | 9 | 20 | 12 | 12 | 20 |
| nuclear periphery | 0 | 6 | 4 | 8 | 4 | 11 | 1056 | 4 | 21 | 4 | 15 | 31 |
| nucleolus | 0 | 2 | 5 | 0 | 0 | 8 | 4 | 1112 | 45 | 0 | 77 | 10 |
| nucleus | 2 | 22 | 4 | 1 | 6 | 18 | 12 | 135 | 1386 | 0 | 21 | 20 |
| peroxisome | 0 | 0 | 2 | 0 | 3 | 6 | 1 | 1 | 1 | 131 | 18 | 1 |
| spindle pole | 2 | 25 | 77 | 1 | 1 | 18 | 33 | 1 | 8 | 18 | 595 | 2 |
| vacuole | 0 | 0 | 3 | 1 | 8 | 11 | 8 | 7 | 3 | 0 | 4 | 542 |

To see misclassification patterns more clearly, the diagonal of the confusion matrix has been excluded in the following figure.

### Protein classification accuracy

Across proteins, the mean and median accuracy were 0.989 and 1.000, respectively.

| Class | Number of proteins | Correct calls | Accuracy |
| --- | --- | --- | --- |
| cell periphery | 7 | 7 | 1.000 |
| cytoplasm | 108 | 107 | 0.991 |
| endosome | 2 | 2 | 1.000 |
| er | 29 | 29 | 1.000 |
| golgi | 2 | 2 | 1.000 |
| mitochondrion | 45 | 45 | 1.000 |
| nuclear periphery | 5 | 5 | 1.000 |
| nucleolus | 7 | 7 | 1.000 |
| nucleus | 69 | 67 | 0.971 |
| peroxisome | 1 | 1 | 1.000 |
| spindle pole | 2 | 2 | 1.000 |
| vacuole | 5 | 5 | 1.000 |

### Protein classification accuracy (at least 10 cell images)

Across proteins with at least 10 cell images, the mean and median accuracy were 1.000 and 1.000, respectively.

| Class | Number of proteins | Correct calls | Accuracy |
| --- | --- | --- | --- |
| cell periphery | 7 | 7 | 1 |
| cytoplasm | 66 | 66 | 1 |
| endosome | 2 | 2 | 1 |
| er | 27 | 27 | 1 |
| golgi | 2 | 2 | 1 |
| mitochondrion | 42 | 42 | 1 |
| nuclear periphery | 5 | 5 | 1 |
| nucleolus | 7 | 7 | 1 |
| nucleus | 57 | 57 | 1 |
| peroxisome | 1 | 1 | 1 |
| spindle pole | 2 | 2 | 1 |
| vacuole | 4 | 4 | 1 |

### Misclassifications - false negatives

Original and scaled cell images, respectively.

```
## [1] "cell periphery, false negative rate: 0.097"
```

```
## [1] "cytoplasm, false negative rate: 0.092"
```

```
## [1] "endosome, false negative rate: 0.351"
```

```
## [1] "er, false negative rate: 0.123"
```

```
## [1] "golgi, false negative rate: 0.152"
```

```
## [1] "mitochondrion, false negative rate: 0.089"
```

```
## [1] "nuclear periphery, false negative rate: 0.093"
```

```
## [1] "nucleolus, false negative rate: 0.12"
```

```
## [1] "nucleus, false negative rate: 0.148"
```

```
## [1] "peroxisome, false negative rate: 0.201"
```

```
## [1] "spindle pole, false negative rate: 0.238"
```

```
## [1] "vacuole, false negative rate: 0.077"
```

### Misclassifications - false positives

```
## [1] "cell periphery"
```

```
## [1] "cytoplasm"
```

```
## [1] "endosome"
```

```
## [1] "er"
```

```
## [1] "golgi"
```

```
## [1] "mitochondrion"
```

```
## [1] "nuclear periphery"
```

```
## [1] "nucleolus"
```

```
## [1] "nucleus"
```

```
## [1] "peroxisome"
```

```
## [1] "spindle pole"
```

```
## [1] "vacuole"
```

### Common mistakes (top 5)

```
## [1] "True class: peroxisome (total images 164), predicted as: spindle pole, count: 18 (11.0% error)"
```

```
## [1] "True class: spindle pole (total images 781), predicted as: endosome, count: 77 (9.9% error)"
```

```
## [1] "True class: nucleus (total images 1627), predicted as: nucleolus, count: 135 (8.3% error)"
```

```
## [1] "True class: endosome (total images 689), predicted as: vacuole, count: 56 (8.1% error)"
```

```
## [1] "True class: endosome (total images 689), predicted as: spindle pole, count: 49 (7.1% error)"
```
