# Supplementary material for: Accurate Classification of Protein Subcellular Localization from High-Throughput Microscopy Images Using Deep Learning
Source: G3 (Bethesda). 2017 Apr 8;7(5):1385–92. doi: 10.1534/g3.116.033654 (PMC5427497; doi:10.1534/g3.116.033654)
Supplement: Supplementary file 17 [file 1385FileS4.zip › FileS4.html]

Visual confusion matrix - 1


```
## [1] "True class: nucleus, predicted as: nucleolus, count: 108"
```

```
## [1] "True class: nucleolus, predicted as: spindle pole, count: 72"
```

```
## [1] "True class: spindle pole, predicted as: endosome, count: 65"
```

```
## [1] "True class: er, predicted as: cell periphery, count: 44"
```

```
## [1] "True class: endosome, predicted as: cytoplasm, count: 38"
```

```
## [1] "True class: nucleolus, predicted as: nucleus, count: 38"
```

```
## [1] "True class: endosome, predicted as: spindle pole, count: 37"
```

```
## [1] "True class: cell periphery, predicted as: er, count: 32"
```

```
## [1] "True class: spindle pole, predicted as: nuclear periphery, count: 31"
```

```
## [1] "True class: er, predicted as: golgi, count: 30"
```

### Visual confusion matrix - 1

### Visual confusion matrix - 2

### Visual confusion matrix - 3
