# Supplementary material for: Accurate Classification of Protein Subcellular Localization from High-Throughput Microscopy Images Using Deep Learning
Source: G3 (Bethesda). 2017 Apr 8;7(5):1385–92. doi: 10.1534/g3.116.033654 (PMC5427497; doi:10.1534/g3.116.033654)
Supplement: Supplementary file 19 [file 1385FileS5.zip › FileS5.html]

Visual confusion matrix - 1


```
## [1] "True class: spindle pole, predicted as: endosome, count: 162"
```

```
## [1] "True class: er, predicted as: cell periphery, count: 146"
```

```
## [1] "True class: endosome, predicted as: mitochondrion, count: 132"
```

```
## [1] "True class: er, predicted as: cytoplasm, count: 118"
```

```
## [1] "True class: vacuole, predicted as: nuclear periphery, count: 100"
```

```
## [1] "True class: nucleolus, predicted as: spindle pole, count: 91"
```

```
## [1] "True class: cell periphery, predicted as: er, count: 88"
```

```
## [1] "True class: nucleus, predicted as: nucleolus, count: 83"
```

```
## [1] "True class: cell periphery, predicted as: cytoplasm, count: 81"
```

```
## [1] "True class: cell periphery, predicted as: golgi, count: 76"
```

### Visual confusion matrix - 1

### Visual confusion matrix - 2

### Visual confusion matrix - 3
