# Supplementary material for: Accurate Classification of Protein Subcellular Localization from High-Throughput Microscopy Images Using Deep Learning
Source: G3 (Bethesda). 2017 Apr 8;7(5):1385–92. doi: 10.1534/g3.116.033654 (PMC5427497; doi:10.1534/g3.116.033654)
Supplement: Supplementary file 21 [file 1385FileS6.zip › FileS6.html]

Class frequencies


### Class frequencies

### Protein counts per class

Each protein was imaged four times.

| Class | Training images | Validation images | Test images |
| --- | --- | --- | --- |
| actin | 6 | 4 | 15 |
| bud neck | 7 | 4 | 4 |
| lipid particle | 11 | 4 | 4 |
| microtubule | 4 | 2 | 4 |

### Sanity check for class overlap

```
## [1] "# test imgs in training set: 0"
```

```
## [1] "# val imgs in training set: 0"
```

```
## [1] "# test imgs in validation set: 0"
```

### Class examples (from training set)

```
## [1] "actin"
```

```
## [1] "bud neck"
```

```
## [1] "lipid particle"
```

```
## [1] "microtubule"
```

### Scaled class examples (from training set)

```
## [1] "actin"
```

```
## [1] "bud neck"
```

```
## [1] "lipid particle"
```

```
## [1] "microtubule"
```
