# Supplementary material for: Accurate Classification of Protein Subcellular Localization from High-Throughput Microscopy Images Using Deep Learning
Source: G3 (Bethesda). 2017 Apr 8;7(5):1385–92. doi: 10.1534/g3.116.033654 (PMC5427497; doi:10.1534/g3.116.033654)
Supplement: Supplementary file 22 [file 1385FileS6.docx]

File S6. Transfer learning data overview. (.zip, 911 KB)

<http://www.g3journal.org/lookup/suppl/doi:10.1534/g3.116.033654/-/DC1/FileS6.zip>
