# Supplementary material for: Accurate Classification of Protein Subcellular Localization from High-Throughput Microscopy Images Using Deep Learning
Source: G3 (Bethesda). 2017 Apr 8;7(5):1385–92. doi: 10.1534/g3.116.033654 (PMC5427497; doi:10.1534/g3.116.033654)
Supplement: Supplementary file 23 [file 1385FileS7.docx]

**Supplementary Figure 1**. Confusion matrices for DeepYeast (a) and random forest (b). The error rates (colour) of predicting the true class (y-axis) to a different class (x-axis) are given for each class pair in the heatmap.

**Supplementary Figure 2**. Number of protein classification errors (y-axis) for different number of recorded cells (x-axis) for DeepYeast (red) and random forest (blue).

**Supplementary Figure 3**. t-SNE projections for each output layer of DeepYeast. 1,000 randomly sampled cell images were processed with DeepYeast, and the neuron outputs at each layer processed with t-SNE to give a two-dimensional representation of the data. The markers are colored according to the 12 compartments, and the panel colors indicate the type of layer (see also Fig. 1c).

**Supplementary Figure 4**. Maximum correlation of a DeepYeast output to Gabor filters (a) and CellProfiler features (b). A random sample of 1,000 cell images was processed with DeepYeast, and the outputs recorded for every neuron at each layer. We calculated Pearson correlation coefficients between each neuron output, and each Gabor filter feature (a) or CellProfiler feature (b) across the 1,000 images. The largest of the correlation coefficients (y-axis) across the neurons is plotted for each layer (x-axis).

**Supplementary Figure 5**. Transfer learning per class. Precision (y-axis, upper panels) and recall (y-axis, lower panels) for the four additional classes (actin, bud neck, lipid particle, and microtubule; left to right) using opimized random forests on CellProfiler features (blue) or DeepYeast fully connected layer outputs (red) for increasing numbers of training images (x-axis).

**Supplementary Figure 6**. Transfer learning per layer. Accuracy (y-axis) for the four additional classes for increasing numbers of training images (x-axis). In all panels, the prediction performance based on raw data (green), CellProfiler features (purple), and DeepYeast class probability (pink) is given as reference. The performance based on neuron output from the first (a), second (b), and third (c) convolutional layers, as well as the fully connected layers (d) is depicted in thin lines for comparison.

**Supplementary Table 1**. Confusion matrices for DeepYeast and random forest on test data.

**Supplementary Table 2**. Precision and recall bootstrap confidence intervals for DeepYeast and random forest on test data.

**Supplementary File 1**. Data overview

**Supplementary File 2**. Classification performance overview for DeepYeast

**Supplementary File 3**. Classification performance overview for random forest

**Supplementary File 4**. Frequent classification mistakes for DeepYeast

**Supplementary File 5**. Frequent classification mistakes for random forest

**Supplementary File 6**. Transfer learning data overview
